# Chaperone-mediated autophagy as a regulator of hallmarks of cancer

**DOI:** 10.3389/fonc.2026.1927298

**Published:** 2026-08-27

**Authors:** Meenakshi Tiwari, Lokendra Kumar Sharma, Bandana Chakravarti, Veena Kumari Singh, Shruti Singh, Ashutosh Shrivastava

**Affiliations:** 1 Center for Advance Research, King George’s Medical University, Lucknow, Uttar Pradesh, India; 2 Department of Molecular Medicine & Biotechnology, Sanjay Gandhi Post Graduate Institute of Medical Sciences, Lucknow, Uttar Pradesh, India; 3 Department of Burns & Plastic Surgery, All India Institute of Medical Sciences, Patna, Bihar, India; 4 Department of Pharmacology, All India Institute of Medical Sciences, Patna, Bihar, India

**Keywords:** autophagy, carcinogenesis, chaperone-mediated autophagy, hallmarks of cancer, proteostasis

## Abstract

Chaperone-mediated autophagy (CMA) is a selective lysosomal degradation pathway that maintains cellular homeostasis by degrading soluble proteins containing KFERQ-like motifs. Although CMA has traditionally been recognized for its role in protein quality control and cellular stress adaptation, increasing evidence shows that it is frequently altered in cancer, where it regulates multiple processes that promote tumor initiation, progression, and therapy resistance. The growing number of identified CMA substrates involved in cell proliferation, apoptosis, metabolism, DNA damage response, immune regulation, inflammation, and cellular plasticity suggests that CMA is much more than a protein degradation pathway; it is an important regulator of tumor adaptation. In this review, we bring together current evidence to provide a comprehensive understanding of how CMA contributes to the *Hallmarks of Cancer*, including sustained proliferative signaling, resistance to cell death, metabolic reprogramming, invasion and metastasis, immune evasion, and the enabling characteristics of genome instability and tumor-promoting inflammation. We further explore the emerging roles of CMA in cellular plasticity and cancer stem cell maintenance, two interconnected processes that drive tumor progression, metastasis, and therapeutic resistance. By integrating evidence from diverse tumor types, this review provides a comprehensive understanding of how CMA shapes multiple hallmarks of cancer by selectively degrading key regulatory proteins. Finally, we highlight the context-dependent roles of CMA, identify key gaps in our current understanding, and discuss the opportunities and challenges of targeting CMA for cancer therapy. Overall, this hallmark-based perspective provides an integrated understanding of how CMA contributes to multiple hallmarks of cancer and supports its potential as a therapeutic target.

## 1 Introduction

Cancer remains one of the leading causes of death worldwide and continues to be a major challenge for modern medicine. Despite substantial advances in surgery, chemotherapy, targeted therapy, and immunotherapy, long-term clinical success remains limited. Therapeutic resistance, tumor recurrence, and metastasis continue to be the major barriers to effective cancer treatment (1). These challenges largely stem from cancer cells’ remarkable ability to survive and adapt under adverse conditions. Throughout tumor progression, cancer cells are exposed to nutrient deprivation, hypoxia, immune surveillance, and therapeutic pressure. Instead of being eliminated, they activate adaptive mechanisms that promote survival and disease progression. These adaptive changes arise through genetic and epigenetic alterations as well as the rewiring of cellular pathways. As a result, cancer cells remodel their metabolism, alter gene expression, evade immune destruction, and maintain protein homeostasis. Together, these changes enable cancer cells to acquire the hallmarks of cancer, including sustained proliferation, resistance to cell death, invasion, metastasis, and long-term survival in hostile tumor microenvironments (2).

A growing body of evidence suggests that the successful acquisition and maintenance of these hallmarks depend heavily on cellular quality-control systems that preserve protein and organelle homeostasis under physiological conditions and during chronic stress. Among these, lysosome-dependent proteolytic pathways have emerged as critical regulators of tumor adaptation by selectively remodeling the cellular proteome in response to metabolic, oxidative, and therapeutic challenges. Within this context, chaperone-mediated autophagy (CMA), the most selective form of autophagy, has emerged as an important regulator of cellular homeostasis and is increasingly recognized for its diverse roles in cancer biology. By selectively degrading specific substrate proteins, CMA influences cellular processes such as metabolic adaptation, stress responses, stemness, immune modulation, invasion, and therapeutic resistance (3). Although these functions have largely been studied independently, they collectively suggest that CMA may contribute to multiple hallmarks of cancer. Accordingly, this review proposes a hallmark-of-cancer–based framework to integrate the diverse roles of CMA and to provide a mechanistic perspective on how this selective autophagic pathway shapes tumor progression, while highlighting its therapeutic potential.

## 2 The hallmarks of cancer framework — an overview

The hallmarks of cancer framework, developed by Douglas Hanahan and Robert Weinberg, provides a conceptual framework with a unifying model describing how diverse cancers develop by progressively acquiring a common set of biological capabilities (4). The original model described six essential biological capabilities acquired by cancer cells during the multistep process of tumorigenesis: sustaining proliferative signaling, evading growth suppressors, resisting cell death, enabling replicative immortality, inducing angiogenesis, and activating invasion and metastasis (4).

**Figure 1:**
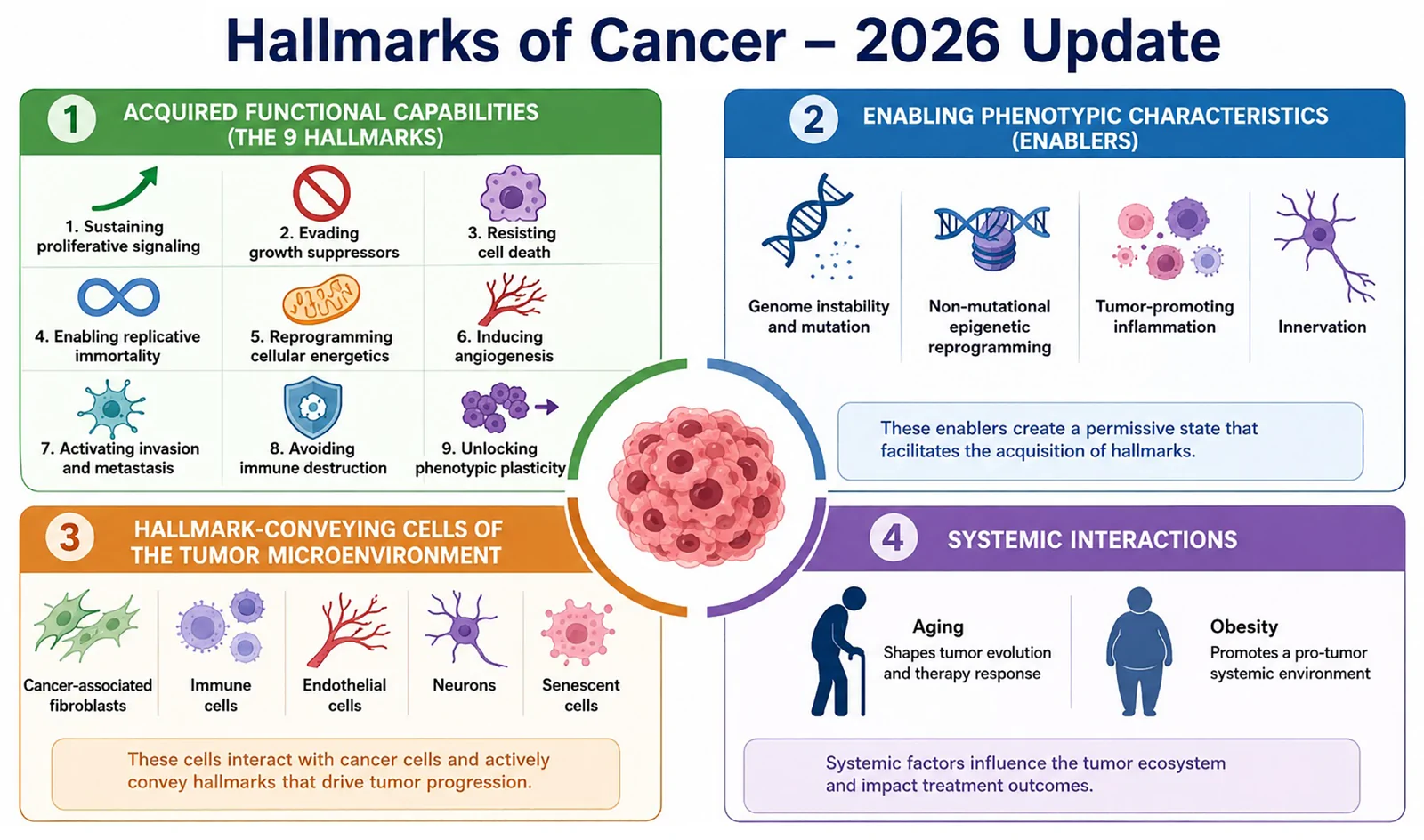
Hallmarks of cancer: four-dimensional framework. The 2026 Hallmarks of cancer framework depicts cancer as a dynamic ecosystem organized into four interconnected dimensions: (1) acquired functional capabilities (the nine hallmarks), (2) enabling phenotypic characteristics (genome instability, epigenetic reprogramming, tumor-promoting inflammation, and innervation), (3) hallmark-conveying cells of the tumor microenvironment, and (4) systemic interactions, including aging and obesity. Together, these dimensions illustrate how intrinsic cellular changes, the tumor microenvironment, and host factors collectively drive tumor initiation, progression, and therapeutic response.

The framework was substantially expanded in the landmark 2011 update, which introduced two additional validated hallmarks — reprogramming of cellular energetics and avoiding immune destruction. It also formally recognized genome instability and mutation, and tumor-promoting inflammation as enabling characteristics that facilitate the acquisition of hallmarks rather than representing hallmarks per se (5). A further major revision in 2022 introduced four emerging hallmarks: unlocking phenotypic plasticity, non-mutational epigenetic reprogramming, the influence of polymorphic microbiomes, and the contribution of senescent cells to tumor progression (6). In the most recent 2026 update, the entire framework was reorganized into four distinct but interconnected parametric dimensions (summarized in **Figure 1**): (i) acquired functional capabilities (the nine hallmarks), (ii) enabling phenotypic characteristics including genome instability, epigenetic reprogramming, tumor-promoting inflammation, and the newly formalized role of innervation, (iii) hallmark-conveying cells of the tumor microenvironment — comprising cancer-associated fibroblasts, immune cells, endothelial cells, neurons, and senescent cells — and (iv) systemic interactions, particularly aging and obesity, which modulate tumor evolution and therapeutic responsiveness (7).

Within this four-dimensional framework, cancer no longer appears as a cell-autonomous disease but as a dynamic ecosystem in which genetic, epigenetic, and environmental forces converge. The ninth hallmark — unlocking phenotypic plasticity — captures the capacity of cancer cells to escape terminal differentiation and adopt stem-like, hybrid epithelial–mesenchymal, or dedifferentiated states, fundamentally complicating therapeutic targeting.

The present review mainly centers on the first dimension of this framework (the nine acquired functional hallmarks); CMA also intersects with the remaining three dimensions, as discussed throughout this review. Current evidence suggests that CMA contributes to hallmark-enabling processes, including the regulation of genome stability and inflammatory signaling, influences both tumor cell-intrinsic functions and the activities of hallmark-conveying cells within the tumor microenvironment, and is subject to regulation by systemic physiological and metabolic conditions that may impact tumor evolution. Recognizing these broader connections provides a more integrated framework for understanding the multifaceted role of CMA in cancer biology, and these aspects are highlighted where relevant throughout the review.

## 3 Chaperone-mediated autophagy: an evolving regulator of cellular homeostasis

**Figure 2:**
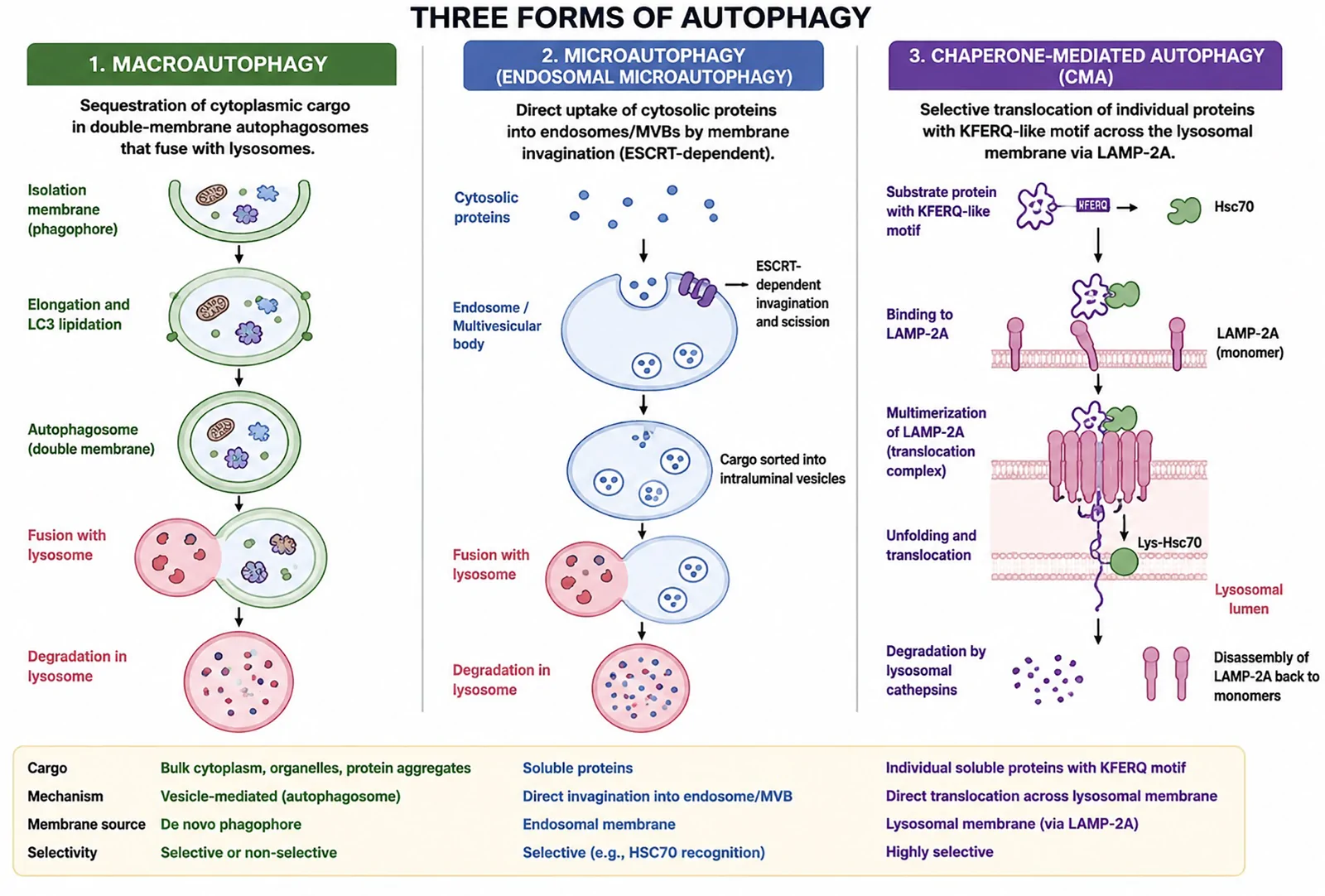
Major autophagic pathways. Macroautophagy sequesters bulk cytoplasmic cargo within double-membrane autophagosomes for lysosomal degradation; microautophagy internalizes cytosolic components through direct endosomal/lysosomal membrane invagination; and chaperone-mediated autophagy (CMA) selectively degrades soluble proteins containing KFERQ-like motifs via Hsc70- and LAMP-2A-mediated translocation across the lysosomal membrane. Together, these pathways maintain cellular proteostasis and metabolic homeostasis through distinct cargo recognition and delivery mechanisms.

Autophagy, a catabolic process, removes damaged and unwanted macromolecules and organelles under physiological and pathological conditions. The three major autophagic pathways—macroautophagy, microautophagy, and CMA—are collectively involved in maintaining cellular proteostasis by ensuring protein quality control through the removal of damaged, misfolded, or excess proteins, thereby preserving metabolic homeostasis (**Figure 2**). These pathways converge on the lysosome as the terminal degradative organelle, yet they differ profoundly in their cargo recognition mechanisms, machinery, selectivity, stress responsiveness, and contributions to cancer biology (8, 9). Macroautophagy, the most extensively studied form of autophagy, is initiated by activation of the ULK1 complex, followed by phagophore nucleation, membrane elongation through ATG protein-mediated LC3 lipidation, and formation of double-membraned autophagosomes that engulf cytoplasmic cargo and fuse with lysosomes for degradation. Although originally regarded as a non-selective bulk degradation pathway, macroautophagy can also selectively eliminate damaged mitochondria (mitophagy), endoplasmic reticulum (ER-phagy), ribosomes (ribophagy), lipid droplets (lipophagy), protein aggregates (aggrephagy), and invading pathogens (xenophagy) through specialized cargo receptors such as p62/SQSTM1, NDP52, OPTN, and NBR1 (10). This remarkable flexibility enables macroautophagy to remove large cytoplasmic structures that cannot be processed by other lysosomal pathways. Macroautophagy is rapidly induced as a primary emergency recycling pathway during acute nutrient deprivation, hypoxia, oxidative stress, and genotoxic injury, providing amino acids and metabolic substrates that sustain cellular survival under conditions of energy shortage (11).

In cancer, macroautophagy plays a context-dependent role, functioning as a tumor suppressor during early tumorigenesis by limiting oxidative stress, genomic instability, and chronic inflammation, while subsequently supporting established tumors by promoting metabolic adaptation, stress survival, therapeutic resistance, and tumor progression (8, 9). Microautophagy, primarily represented in mammalian cells by endosomal microautophagy, mediates the direct uptake of cytosolic proteins into late endosomes or multivesicular bodies through ESCRT-dependent inward membrane invagination and vesicle scission. Selective cargo recognition is frequently mediated by Heat shock cognate protein 70 (HSC70), which binds KFERQ-like motifs and facilitates cargo delivery to endosomal membranes for lysosomal degradation or sorting into intraluminal vesicles. This pathway has emerged as an important regulator of protein quality control, nutrient adaptation, and intercellular communication through the endosomal–exosomal axis (12). Comprehensive discussions of the molecular mechanisms and cancer-related functions of macroautophagy and microautophagy are available in several recent reviews and are therefore not covered in detail here (8, 9, 12, 13).

Among these lysosomal pathways, CMA is distinguished by its exceptional substrate specificity: rather than engulfing bulk cytoplasm or organelles, it selects individual proteins bearing a KFERQ-like pentapeptide motif (Lysine-K, Phenylalanine-F, Glutamic Acid-E, Arginine-R, Glutamine-Q), present in roughly 30-40% of cytosolic proteins, for direct translocation across the lysosomal membrane. Unlike the largely non-selective, bulk-degradative flux of macroautophagy or the membrane-invagination-based cargo capture of microautophagy, CMA operates continuously at basal rates and is further inducible under prolonged starvation, oxidative stress, and proteotoxic insult, allowing its flux to be dynamically tuned to cellular demand rather than triggered as an all-or-none event (14, 15).

CMA is a cargo-based, highly selective lysosomal degradation pathway that translocates specific soluble proteins across the lysosomal membrane without vesicle formation. The process initiates when the cytosolic chaperone Hsc70, together with its co-chaperones HSP40, HSP90, HSP70-interacting protein (HIP), HSP70-HSP90 organizing protein (HOP), and Bcl-2-associated athanogene (BAG) family proteins, recognizes a KFERQ-like motif within the substrate protein (16). This chaperone-substrate complex then targets the lysosomal membrane, where the substrate binds specifically to the cytosolic tail of the monomeric receptor Lysosome-Associated Membrane Protein 2A (LAMP-2A) (17). This binding event triggers the multimerization of LAMP-2A into a high-molecular-weight translocation channel. To transition through this narrow pore, the substrate must undergo mandatory unfolding, a process driven by cytosolic chaperones. The unfolded polypeptide is then pulled through the channel into the lysosomal lumen, a directional translocation facilitated by a resident lumenal chaperone, lys-Hsc70. Once inside, the substrate is rapidly degraded into amino acids by hydrolytic cathepsins, while the LAMP-2A complex is disassembled back into monomers to reset the pathway (15). Unlike conventional receptors, LAMP-2A does not function as a static docking protein. Instead, its abundance and dynamic assembly at the lysosomal membrane largely determine overall CMA activity, making LAMP-2A the principal regulator of CMA flux (17). Moreover, CMA is tightly regulated at multiple levels to ensure that protein degradation matches cellular demand. LAMP-2A expression, lysosomal membrane stability, HSC70 availability, lysosomal pH, and ATP levels collectively determine CMA activity. In addition, signaling pathways including mTORC2-AKT-PHLPP1, retinoic acid receptor-α (RARα), calcineurin-NFAT signaling, and oxidative stress-responsive pathways dynamically regulate LAMP-2A assembly or stability, allowing rapid modulation of CMA flux without requiring *de novo* synthesis of the entire degradative machinery (18–21). This multilayered regulation enables cells to degrade/regulate selected proteins in response to metabolic and environmental signals, distinguishing CMA from the more generalized activation mechanisms governing macroautophagy (22).

Under physiological conditions, CMA fulfills essential homeostatic functions across multiple biological contexts (14). It contributes to protein quality control by selectively clearing oxidatively damaged, misfolded, or functionally surplus proteins that would otherwise seed toxic aggregates. CMA participates in metabolic regulation by mediating the timed degradation of key enzymes in glycolysis, lipid metabolism, and one-carbon metabolism, thereby enabling rapid metabolic adaptation to changes in nutrient availability (23). CMA is activated following T-cell receptor (TCR) stimulation, promoting T-cell activation by selectively degrading negative regulators, such as ITCH (Itchy E3 ubiquitin protein ligase) and RCAN1 (Regulator of Calcineurin 1). Age-associated decline in CMA impairs T-cell function, whereas restoration of LAMP-2A enhances immune responses in aged T cells (20). Further, CMA also contributes to antigen presentation and innate immune signaling (14, 20, 24, 25). Beyond protein quality control, CMA regulates cell-cycle progression by selectively degrading Checkpoint Kinase 1 (CHK1) and preserves stem cell homeostasis by controlling the proteostasis of metabolic enzymes that orchestrate metabolic reprogramming and differentiation (26–28). With age, CMA activity declines progressively, primarily due to destabilization of LAMP2A within lysosomal lipid microdomains. This leads to accumulation of damaged proteins, impaired stress responses, and metabolic dysfunction — a phenotype rescued by transgenic maintenance of LAMP2A levels in aged rodents (29).

Importantly, CMA does not operate in isolation; extensive functional crosstalk and compensatory interactions exist among CMA, macroautophagy, and microautophagy, enabling cells to adapt dynamically to diverse stresses. In cirrhotic liver and hepatocellular carcinoma, for example, chronic impairment of macroautophagic flux is functionally compensated by induction of CMA, and more than 95% of hepatocellular carcinomas show altered LAMP-2A expression relative to surrounding cirrhotic tissue, underscoring that the two pathways are reciprocally, not independently, regulated in the tumor setting (30). This compensatory relationship has direct implications for CMA-targeted therapy, since pharmacological inhibition of CMA alone may be buffered by compensatory upregulation of macroautophagy, and combined inhibition of both pathways has been shown to more effectively suppress tumor growth than inhibition of either pathway alone (31). A further, less appreciated layer of regulation arises from substrate competition: because KFERQ-like motifs are present in a substantial fraction of the cytosolic proteome, individual substrates compete for a limiting pool of HSC70 and LAMP-2A translocation complexes, so that changes in the abundance of one CMA substrate can indirectly alter the degradation kinetics of others, a phenomenon with direct consequences for interpreting substrate-specific phenotypes in cancer models (32, 33). However, the exceptional specificity of CMA allows it to directly regulate the stability of numerous oncogenic proteins, tumor suppressors, metabolic enzymes, transcription factors, and immune regulators. As a result, CMA occupies a unique position to regulate multiple dimensions of the updated Hallmarks of Cancer framework.

## 4 CMA as a mediator of tumorigenesis

Over the past decade, CMA has emerged from being viewed primarily as a protein quality-control pathway to being recognized as a context-dependent regulator of tumor biology, capable of both pro-tumorigenic and tumor-suppressive functions depending on tumor type, genetic background, and disease stage. The importance of CMA-mediated regulation in cancer was first demonstrated by Kon et al. (34), whose landmark study fundamentally changed the understanding of CMA in tumor biology (34). Using multiple transformed cell lines, primary human tumors, and xenograft models, the authors showed that CMA activity is constitutively elevated in malignant cells compared with their non-transformed counterparts. More importantly, silencing the CMA receptor LAMP-2A did not simply impair protein degradation but profoundly disrupted the malignant phenotype by suppressing glycolytic metabolism, reducing ATP production, stabilizing wild-type p53, impairing proliferation and metastatic capacity, and markedly inhibiting tumor growth *in vivo*. These findings established that cancer cells develop a functional dependence on sustained CMA activity, suggesting that CMA is not merely activated during transformation but becomes an essential component of the malignant state (34). Subsequent studies revealed that this dependence is driven by the selective degradation of proteins governing diverse oncogenic processes. One of the earliest mechanistic examples was provided by Lv et al. (35), who demonstrated that CMA regulates metabolic reprogramming and glucose utilization in cancer cells (discussed in the following sections) (35). Shortly thereafter, Gomes et al. reported that CMA promotes the proteasomal degradation of the proto-oncogene MYC, preventing malignant transformation under physiological conditions (36). Together, these studies established an important conceptual principle: the biological outcome of CMA is determined not by the pathway itself but by the identity of the substrates it selectively degrades. Depending on the cellular context, CMA can therefore either eliminate oncogenic proteins or remove tumor-suppressive regulators, resulting in diametrically opposite effects on tumor progression.

The context-dependent nature of CMA has been demonstrated across multiple tumor models. In cirrhotic liver, where macroautophagy is chronically impaired, Chava et al. showed that compensatory activation of CMA promotes hepatocellular carcinoma (HCC), with altered LAMP-2A expression detected in more than 95% of human HCC specimens. Genetic inhibition of LAMP-2A significantly reduced cell proliferation, migration, colony formation, and tumorigenicity, demonstrating that tumor cells exploit CMA as an adaptive survival mechanism when macroautophagy is compromised (30). Similarly, Li et al. reported that glioblastoma stem cells (GSCs) are highly dependent on elevated CMA activity to maintain stemness, metabolic fitness, and immune evasion. LAMP-2A inhibition impaired self-renewal, restored anti-tumor immunity, and suppressed tumor growth, identifying CMA as a critical regulator of cancer stem cell maintenance (31). Extending these findings, Zhou et al. demonstrated that CMA governs the metastatic state of mesenchymal tumors by selectively remodeling proteins involved in cellular plasticity and invasion, establishing CMA as an active driver of metastatic competence rather than merely a stress-response pathway (37).

However, accumulating evidence indicates that CMA cannot be regarded solely as a tumor-promoting pathway. Instead, its biological effects are dictated by tumor type, disease stage, and, most importantly, the repertoire of substrates targeted for degradation. For example, it was demonstrated that CMA activation suppresses glioblastoma progression by selectively degrading wild-type IDH1, thereby disrupting metabolic programs required for tumor growth (38). Likewise, CMA was identified as a tumor-suppressive mechanism in HCC, demonstrating that its restoration restrained malignant progression (39). Additional substrate-specific studies reinforce this concept: CMA-mediated degradation of RND3 promotes gastric cancer progression (40), degradation of p21 facilitates colorectal tumor growth (40), whereas degradation of the pro-apoptotic proteins PUMA and p73 suppresses apoptosis and enhances cancer cell survival (41, 42). These findings demonstrate that the consequences of CMA activation cannot be generalized as uniformly oncogenic or tumor suppressive; rather, CMA functions as a substrate-selective proteostasis regulator, with its biological output determined by the cellular context and the proteins targeted for degradation.

Collectively, these findings establish that the biological impact of CMA extends far beyond protein quality control. By selectively regulating the stability of oncogenes, tumor suppressors, metabolic enzymes, DNA damage regulators, and immune modulators, CMA dynamically remodels multiple signaling networks at the post-translational level without altering the underlying genome. Consequently, dysregulated CMA influences diverse aspects of tumor biology in a highly context- and substrate-dependent manner, providing a mechanistic basis for its involvement in virtually every cancer hallmark (8, 37–39). By selectively degrading proteins that restrain proliferation, enforce DNA damage checkpoints, promote apoptosis, maintain genomic integrity, and license innate immune surveillance, the HSC70–LAMP2A axis mechanistically engages nearly every hallmark capability and enabling characteristic defined in the updated Hanahan framework (4–7).

In the following sections, we systematically examine the contribution of CMA to each hallmark capability and enabling characteristic of this framework, synthesizing evidence from cell lines, patient-derived models, xenograft studies, and TCGA-based bioinformatics analyses. The review contextualizes CMA’s unique position in tumorigenesis and identifies its therapeutic implications. We have not discussed the substrates of CMA involved in hallmarks separately; however, interested readers may refer to the review by Rios et al. (43). Together, the evidence reviewed here positions CMA not as a single-function degradative pathway, but as one of the master proteostatic rheostats whose systematic dysregulation in cancer cells drives coordinated engagement of virtually every facet of the malignant phenotype. The following sections, therefore, focus specifically on how dysregulated CMA contributes to the acquisition and maintenance of the hallmarks of cancer.

## 5 CMA contributes to the hallmarks of cancer

**Figure 3:**
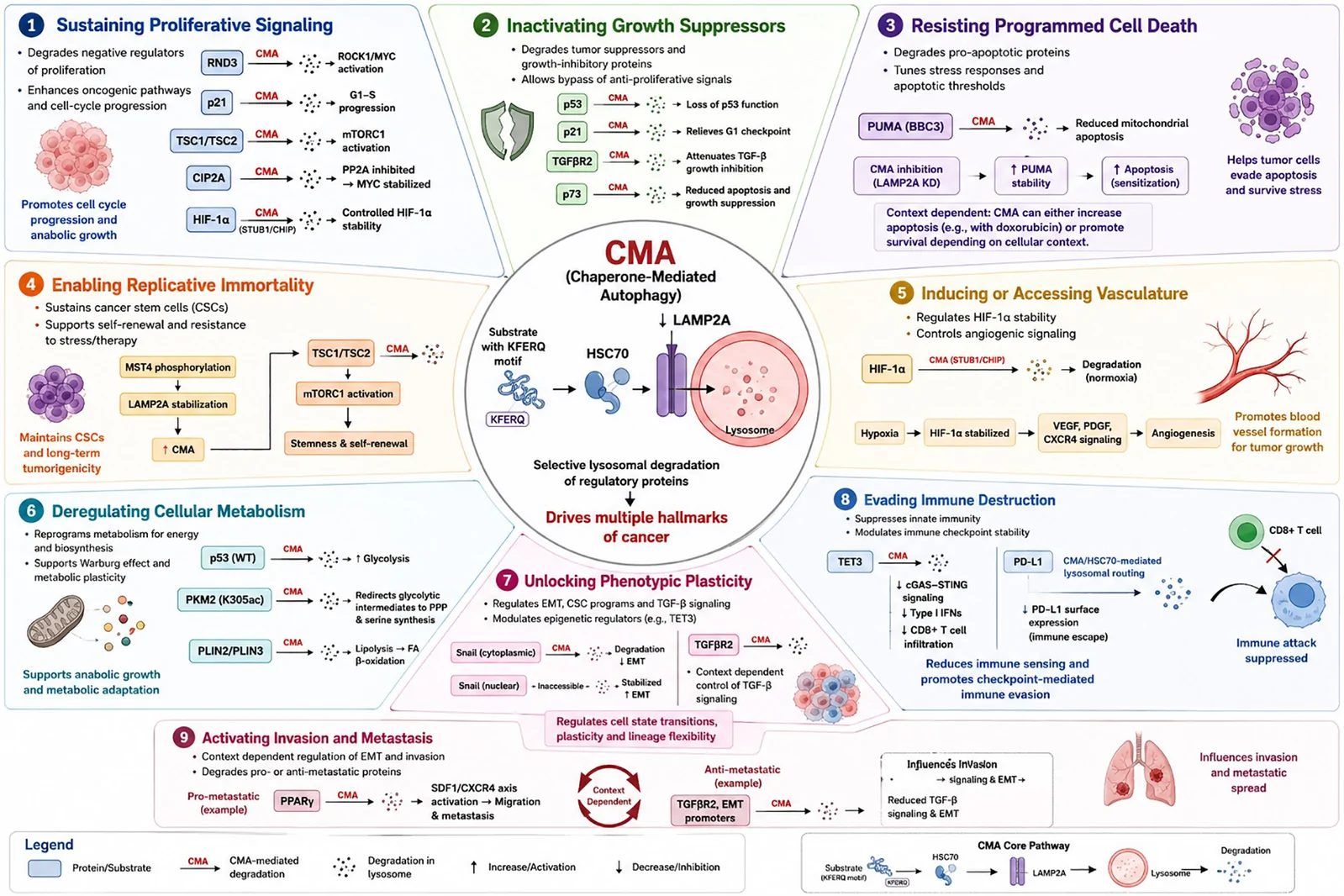
CMA as a regulator of the hallmarks of cancer. CMA selectively degrades KFERQ motif-containing proteins through the HSC70–LAMP-2A lysosomal pathway, thereby influencing multiple canonical and emerging hallmarks of cancer. By controlling the turnover of key regulatory proteins, CMA sustains proliferative signaling, attenuates growth-suppressive pathways, modulates apoptotic responses, supports cancer stem cell maintenance and replicative immortality, regulates angiogenesis and metabolic reprogramming, promotes phenotypic plasticity and immune evasion, and exerts context-dependent effects on invasion and metastasis. Collectively, CMA functions as a central proteostatic regulator that integrates stress adaptation, metabolism, signaling, and cell fate decisions to drive tumor progression and represents a promising therapeutic target across diverse malignancies. In this schematic, red arrows denote activation, stabilization, or promotion of the indicated hallmark process, whereas blue T-shaped (blunt-ended) lines denote inhibition, degradation, or suppression of the indicated process; substrates and hallmark outcomes for which CMA exerts a tumor-suppressive (rather than pro-tumorigenic) effect, such as STAG2 in hepatocellular carcinoma and TGFβR2-dependent EMT restraint in mesenchymal tumors, are indicated separately to reflect the context-dependent directionality of CMA action.

The framework proposed by Hanahan offers a useful framework for organizing the many pro-tumorigenic roles of CMA. In the most recent revision of the Hallmarks of Cancer, Hanahan explicitly introduced “pro‑survival autophagy” and “unlocking phenotypic plasticity” as emerging hallmarks, thereby formally embedding lysosomal pathways such as CMA within the core conceptual architecture of cancer biology (7). This updated framework provides an important rationale for viewing CMA not merely as an auxiliary stress-response mechanism but as a key driver that intersects with multiple canonical and emerging hallmarks across diverse tumor types. The contribution of CMA to each hallmark is discussed in the following sections, and the major mechanisms through which CMA influences these hallmarks are summarized in **Figure 3** and **Table 1**.

**Table 1:** CMA substrates and its role in regulation of different hallmarks of cancer.

Hallmark	CMA substrate/target	Direct or indirect	Role in the main pathway involved	Ref.
Sustaining proliferative signaling	RND3 (RhoE)	Direct CMA substrate	Degradation releases ROCK1 signaling, enhances MYC activity, promotes G1/S progression in gastric cancer.	(44)
	p21 (CDKN1A)	Direct CMA substrate	SNX10-regulated CMA degradation removes G1/S checkpoint restraint, sustains colorectal cancer proliferation.	(40)
	TSC1/TSC2 complex	Direct CMA substrate	MST4-stabilized LAMP-2A enhances CMA, degrading TSC1/TSC2 and activating mTORC1-S6K1/4EBP1 signaling in GSCs.	(31)
	CIP2A (CIP2A-PP2A-MYC axis)	Indirect CMA-regulated target	CMA degrades CIP2A, permitting PP2A-mediated MYC destabilization; CMA loss stabilizes MYC and drives CDK4/CCND1 expression.	(36, 45)
	HIF-1α	Direct CMA-associated substrate	STUB1/CHIP-dependent lysosomal routing limits hypoxia-independent HIF-1α activity, constraining pro-angiogenic/growth outputs.	(46)
Evading/inactivating growth suppressors	Wild-type p53	Direct CMA substrate	Degradation relieves repression of glycolytic genes and abolishes p53-mediated cell-cycle arrest, apoptosis and senescence.	(34)
	CHK1	Direct CMA substrate	Degradation after DNA repair facilitates checkpoint recovery; CMA loss causes CHK1 accumulation and prolonged arrest.	(27)
	p21 (CDKN1A)	Direct CMA substrate	Degradation disables checkpoint effector within p53-CHK1-p21-RB network, facilitating G1/S transition in colorectal cancer.	(40)
	TGFβR2	Direct CMA substrate	Turnover attenuates growth-inhibitory TGF-beta signaling in primary tumors; context-dependent limiting of EMT in advanced cancers.	(37, 45)
	p73	Direct CMA substrate	NGFR-driven degradation suppresses p73 transcriptional program and tumor-suppressive function in neuroblastoma.	(41)
Resisting programmed cell death	BBC3/PUMA	Direct CMA substrate	HSC70-LAMP2A-mediated degradation suppresses mitochondrial apoptotic signaling and raises the apoptotic threshold.	(42)
	Wild-type p53	Direct CMA substrate	Drug-induced CMA upregulation accelerates p53 clearance, contributing to acquired chemoresistance.	(26, 34)
	CHK1	Direct CMA substrate	Chemotherapy-induced CMA flux accelerates CHK1 turnover, aiding survival after genotoxic stress.	(27, 34)
Enabling replicative immortality	TSC1/TSC2 complex	Direct CMA substrate	MST4-LAMP2A-driven CMA degrades TSC1/TSC2, sustaining mTORC1-dependent GSC self-renewal.	(31)
	GAPDH	Direct CMA substrate	CMA clearance of oxidatively damaged GAPDH maintains metabolic fitness and stress tolerance in breast cancer cells.	(47)
	Stemness-associated proteome/CSC program	Indirect CMA-dependent program	Elevated CMA flux preserves cancer stem cell self-renewal, tumor-initiating potential and therapy resistance.	(31, 48, 49)
Inducing or accessing vasculature	HIF-1α	Direct CMA-associated substrate	STUB1/CHIP-assisted K63-linked ubiquitination routes HIF-1α for lysosomal degradation under normoxia, limiting VEGF-driven angiogenesis; hypoxia escapes this control.	(46, 50)
Deregulating cellular metabolism	Wild-type p53	Direct CMA substrate	Degradation relieves repression of glycolytic genes, favoring the Warburg shift.	(34)
	PKM2	Direct CMA substrate	Lys305 acetylation generates a KFERQ-like motif; CMA degradation diverts glycolytic intermediates into anabolic pathways (PPP, serine synthesis).	(35)
	PLIN2	Direct CMA substrate	Degradation of this lipid-droplet coat protein initiates lipolysis and fatty-acid mobilization.	(51, 52)
	PLIN3	Direct CMA substrate	Degradation enables lipid-droplet turnover and beta-oxidation during metabolic stress.	(51)
	TGFβR2 (via G6PD)	Indirect, context-reversed	In mesenchymal tumors, CMA loss (not gain) drives TGFβR2-mediated G6PD activation, fueling anabolic/mitochondrial metabolism.	(37)
Unlocking phenotypic plasticity	Snail (SNAI1)	Direct CMA substrate	Cytoplasmic Snail is degraded via HSC70-CMA, restraining EMT competence; nuclear Snail is protected.	(53, 54)
	TSC1/TSC2 complex	Direct CMA substrate	Sustained mTORC1 activity via TSC1/TSC2 degradation preserves stemness-associated plasticity programs in GSCs.	(31, 48, 49)
	TGFβR2	Direct CMA substrate	CMA-regulated TGFβR2 levels position HSC70-LAMP2A at a decision point for epithelial vs plasticity-enabling transitions.	(37)
	TET3	Indirect (epigenetic regulator)	CMA-mediated TET3 turnover links proteostatic control to non-mutational epigenetic reprogramming and lineage plasticity.	(31)
Evading immune destruction	TET3	Direct CMA substrate	MST4-stabilized LAMP2A/CMA degrades TET3, epigenetically silencing cGAS-STING and reducing CD8+ T-cell recruitment in GSCs.	(31)
	ITCH, RCAN1	Direct CMA substrates	TCR-stimulated CMA degrades these negative regulators, promoting T-cell activation (physiological, non-tumor-intrinsic role).	(20)
	PD-L1	Indirect/CMA-associated	HSC70-guided lysosomal degradation (disrupting CMTM6-PD-L1 recycling) lowers checkpoint abundance and can restore anti-tumor T-cell activity.	(55–57)
	STING/TBK1	Direct CMA substrate	LAMP2A-dependent turnover dampens type I interferon signaling; diclofenac-mediated LAMP2A blockade synergizes with anti-PD-1 therapy.	(58)
	LAMP2A (Treg-intrinsic)	Pathway-level, stromal/immune	High basal CMA in regulatory T cells sustains their suppressive proteome and immune tolerance function, indirectly supporting tumor immune evasion.	(59, 60)
Activating invasion and metastasis	PPARγ	Direct CMA substrate	Degradation activates the SDF1/CXCR4 axis, enhancing migration and lymph-node metastasis in papillary thyroid carcinoma.	(61)
	TGFβR2	Direct CMA substrate	In mesenchymal tumors, CMA-mediated TGFβR2 degradation restrains TGF-beta signaling, EMT, dissemination and metastasis (anti-metastatic role).	(37)
	Snail (SNAI1)	Direct CMA substrate	Cytoplasmic Snail turnover regulates EMT competence and metastatic behavior in a context-dependent manner.	(53, 54)
Tumor-promoting inflammation (enabling characteristic)	Macrophage CMA machinery/HIF-1α axis	Indirect CMA-dependent program	Myeloid LAMP2A loss elevates HIF-1α, driving VEGFA/IL-1β secretion and colitis-associated tumorigenesis.	(62)

### 5.1 Hallmark I: sustaining proliferative signaling

One of the defining capabilities of cancer cells is the ability to sustain proliferative signaling independent of external growth cues. CMA contributes to this hallmark by selectively degrading proteins that restrain cell-cycle progression or inhibit oncogenic signaling pathways. Through this targeted proteostasis, CMA modulates proliferative networks that support continuous tumor cell division.

A clear example is observed in gastric cancer, where CMA promotes proliferation by degrading the small GTPase Rho Family GTPase 3 (RND3), a negative regulator of Rho-associated coiled-coil-containing protein kinase 1 (ROCK1) signaling. Loss of RND3 enhances MYC proto-oncogene, bHLH transcription factor (MYC)-dependent transcription and facilitates G1–S phase progression, thereby supporting tumor growth (44).

A similar CMA-dependent mechanism operates in colorectal cancer, where the tumor suppressor sorting nexin-10 (SNX10) regulates CMA-mediated degradation of the cyclin-dependent kinase inhibitor p21^Cip1/WAF1^. Loss of SNX10 enhances CMA activity and accelerates p21 turnover, relieving the G1 checkpoint and promoting sustained cell-cycle progression (15, 40, 63).

CMA also supports proliferative signaling in stem-like tumor populations. In glioblastoma stem cells, Mammalian STE20-like protein kinase 4 (MST4) phosphorylates and stabilizes the lysosomal receptor LAMP-2A, thereby enhancing CMA activity. Increased CMA promotes degradation of the tumor suppressor complex Tuberous Sclerosis Complex 1/2 (TSC1/TSC2), leading to constitutive activation of mechanistic Target of Rapamycin Complex 1 (mTORC1), which drives ribosome biogenesis, cap-dependent translation, and ribosomal protein S6 kinase 1 (S6K1)–eukaryotic translation initiation factor 4E-binding protein 1 (4EBP1) signaling, thereby supporting the biosynthetic demands of rapidly proliferating cells (31).

Another regulatory mechanism involves the CMA–Cancerous Inhibitor of Protein Phosphatase 2A (CIP2A)–Protein Phosphatase 2A (PP2A)–MYC axis. Under normal conditions, CMA degrades the oncoprotein CIP2A, which otherwise inhibits PP2A. This allows PP2A-mediated destabilization of MYC, thereby limiting MYC-driven transcription. Conversely, CMA activity is reduced, CIP2A accumulates, PP2A remains inhibited, and MYC becomes stabilized, promoting expression of pro-proliferative genes such as Cyclin-Dependent Kinase 4 (CDK4) and Cyclin D1 (CCND1) (36, 45).

Beyond direct regulation of cell-cycle and MYC signaling, CMA also influences proliferative signaling by controlling the stability of hypoxia-responsive transcription factors. CMA-related control of hypoxia-inducible factor 1α (HIF-1α) stability via STIP1 homology and U-Box containing protein 1 (STUB1)/C-terminus of Hsc70-interacting protein (CHIP)-dependent lysosomal routing provides an additional proliferative checkpoint, limiting inappropriate hypoxia-independent HIF-1α activity and thereby constraining coupled pro-angiogenic and growth-promoting transcriptional outputs (46). Together, these studies demonstrate that CMA sustains tumor cell proliferation by regulating the stability of proteins controlling cell-cycle progression, oncogenic transcription, hypoxia-responsive signaling, and anabolic growth pathways, thereby integrating multiple proliferative circuits that support continuous tumor growth.

### 5.2 Hallmark II: inactivating growth suppressors

Normal cells restrict uncontrolled proliferation through an integrated network of tumor suppressors and cell-cycle checkpoint regulators, including p53, p21, RB, p27, and the DNA damage response. By selectively degrading components of these pathways, CMA can weaken growth-inhibitory signaling and enable tumor cells to bypass cell-cycle checkpoints, thereby sustaining proliferation. Earlier evidence came from Kon et al. (34), who identified wild-type p53 as a functional CMA substrate in transformed cells. The authors showed that constitutively elevated CMA activity promotes lysosomal degradation of p53, thereby relieving p53-mediated repression of glycolytic genes while simultaneously abolishing its ability to induce cell-cycle arrest, apoptosis, and senescence. Genetic inhibition of the CMA receptor LAMP-2A stabilized p53, impaired tumor cell proliferation, and suppressed xenograft growth, establishing CMA-mediated p53 degradation as a critical mechanism by which cancer cells evade growth suppression. Further, CHK1, a key DNA damage checkpoint kinase that coordinates cell-cycle arrest and DNA repair, has been identified as a physiological CMA substrate (34). Park et al. (27) demonstrated that CMA-mediated degradation of CHK1 facilitates checkpoint recovery following DNA repair, whereas impaired CMA results in CHK1 accumulation and prolonged checkpoint activation, highlighting CMA as an important regulator of cell-cycle progression (27). Because CHK1 functions in concert with the p21–RB axis to prevent inappropriate cell-cycle progression after DNA damage, its selective degradation by CMA provides an additional mechanism through which tumor cells may overcome growth-inhibitory checkpoints.

Further evidence for the role of CMA in cell-cycle regulation comes from colorectal cancer, where Zhang et al. (32) demonstrated that CMA promotes degradation of the cyclin-dependent kinase inhibitor p21^Cip1/WAF1^, thereby facilitating G1/S transition and sustained proliferation. Restoration of p21 following CMA inhibition significantly reduced colorectal cancer cell growth, indicating that p21 is a functionally important downstream target of CMA in this context (40). Collectively, these findings indicate that CMA promotes evasion of growth suppression not by targeting a single tumor suppressor, but by coordinately regulating multiple nodes within the p53–CHK1–p21–RB checkpoint network. By selectively degrading these regulatory proteins, CMA lowers the threshold for cell-cycle progression and enables sustained proliferation despite intrinsic anti-proliferative signals.

CMA also modulates transforming growth factor-β (TGF-β) signaling, a key pathway that normally suppresses epithelial cell proliferation during early tumorigenesis. CMA-dependent degradation of TGF-β receptor 2 (TGFβR2) attenuates TGF-β-mediated growth inhibition in primary tumors, thereby facilitating escape from growth inhibition. Because TGF-β exerts stage-specific functions during tumor progression, this same mechanism can also limit TGF-β-driven epithelial–mesenchymal transition (EMT) in advanced cancers, illustrating the context-dependent effects of CMA in tumor progression (37, 45). Additionally, in neuroblastoma, nerve growth factor receptor signaling promotes CMA-dependent degradation of p73, a member of the p53 family with well-established roles in cell-cycle arrest and apoptosis, thereby suppressing its transcriptional program and diminishing its tumor-suppressive functions (41).

Collectively, these studies demonstrate that CMA dismantles growth-suppressive networks at multiple regulatory nodes rather than through a single substrate. By coordinately modulating the p53, CHK1, p21, TGF-β, and p73 pathways in a context-dependent manner, CMA lowers the threshold for cell-cycle progression and facilitates persistent tumor growth despite intrinsic anti-proliferative signals.

### 5.3 Hallmark III: resisting programmed cell death

A central requirement for tumor progression is the ability to evade programmed cell death despite oncogenic stress, metabolic imbalance, or exposure to cytotoxic therapies. CMA contributes to this hallmark by selectively degrading regulators of apoptotic signaling, thereby modulating the balance between pro-survival and pro-cell-death pathways. Experimental suppression of CMA through LAMP2A knockdown increases apoptosis in several cancer models, including colon carcinoma, indicating that CMA activity itself can regulate apoptotic thresholds by controlling the stability of multiple stress-response proteins (64).

One of the clearest mechanistic examples involves the degradation of the pro-apoptotic BH3-only protein PUMA (BBC3). PUMA contains a CMA-recognition motif and can be selectively targeted to lysosomes through the HSC70–LAMP2A pathway. CMA-mediated degradation of PUMA suppresses mitochondrial apoptotic signaling, whereas inhibition of CMA stabilizes PUMA and enhances apoptotic sensitivity. These findings demonstrate that CMA can directly suppress intrinsic apoptosis by eliminating key pro-apoptotic mediators (42).

CMA can also influence therapy-induced apoptosis in a context-dependent manner. In breast cancer cells treated with doxorubicin, active CMA was associated with increased apoptotic sensitivity, whereas silencing of the CMA receptor LAMP2A reduced drug-induced apoptosis and promoted chemoresistance. These findings suggest that although CMA frequently promotes tumor cell survival, its effects on apoptosis are context dependent, and under specific therapeutic conditions, CMA may facilitate apoptotic responses by modulating stress-response pathways (65).

Beyond these substrate-specific mechanisms, CMA also modulates cellular stress responses, including endoplasmic reticulum (ER) stress and oxidative stress, thereby indirectly influencing the activation of intrinsic and extrinsic apoptotic pathways under therapy-induced stress. As such, CMA functions as a dynamic rheostat of apoptotic sensitivity, integrating metabolic and proteotoxic cues to determine whether cancer cells survive or succumb to cytotoxic insults. Importantly, chemotherapeutic stress itself is a potent physiological activator of CMA. Genotoxic and proteotoxic stress induced by cytotoxic agents upregulates LAMP-2A expression and HSC70 activity as part of an adaptive stress response. This treatment-induced increase in CMA accelerates the degradation of pro-apoptotic substrates, including PUMA, CHK1, and wild-type p53, thereby promoting acquired chemoresistance through proteostatic adaptation rather than constitutive CMA activation (26, 27, 34, 42). These findings also have potential therapeutic implications. Because CMA flux increases during and after chemotherapy exposure, combining cytotoxic agents with appropriately timed CMA inhibition, rather than continuous inhibition, may more effectively preserve pro-apoptotic checkpoints and enhance therapeutic sensitivity.

Collectively, these findings demonstrate that CMA regulates apoptotic susceptibility through selective degradation of proteins controlling mitochondrial apoptosis, cellular stress responses, and therapy-induced signaling. Depending on the cellular context and therapeutic stimulus, CMA may either promote tumor cell survival or facilitate apoptosis, highlighting its context-dependent role in determining treatment response.

### 5.4 Hallmark IV: enabling replicative immortality

A defining feature of many tumors is the presence of cancer stem cells (CSCs)—a small subpopulation with long-term self-renewal capacity that sustains tumor growth, therapy resistance, and relapse. Although replicative immortality is classically associated with telomere maintenance, telomerase activation, and escape from senescence, accumulating evidence suggests that it also depends on the long-term persistence of self-renewing CSCs. Current evidence indicates that CMA contributes to this hallmark primarily by preserving CSC survival, self-renewal, and functional fitness, rather than by directly regulating telomere biology.

Compelling evidence comes from studies of GSCs. Using a KFERQ-Dendra CMA reporter, Auzmendi-Iriarte and colleagues demonstrated that CMA activity is significantly higher in GSCs compared with differentiated glioma cells. Genetic inhibition of CMA through LAMP2A knockdown impaired proliferation and self-renewal, increased apoptosis, and delayed tumor growth in orthotopic xenograft models. Consistently, analysis of patient datasets showed that high LAMP2 expression correlates with higher tumor grade, temozolomide resistance, and reduced overall survival, indicating that CMA activity contributes to maintenance of the glioblastoma stem cell compartment (48, 49). Subsequent mechanistic work further clarified how CMA sustains GSC stemness. Li and colleagues showed that the kinase MST4 phosphorylates LAMP2A, stabilizing the receptor and enhancing CMA activity in GSCs. Increased CMA promotes the degradation of the tumor suppressor complex TSC1/TSC2, leading to constitutive activation of mTORC1 signaling, which drives anabolic metabolism and stemness-associated transcriptional programs. Importantly, combined inhibition of CMA and macroautophagy significantly suppressed glioblastoma xenograft growth, highlighting the cooperative role of autophagy pathways in maintaining CSC survival (31).

Evidence from other tumor types also supports a role for CMA in sustaining tumor cell survival and stress tolerance. In breast cancer, LAMP2A expression is elevated in tumor tissues, and increased CMA activity protects cells from oxidative stress. Conversely, inhibition of LAMP2A leads to the accumulation of CMA substrates, such as GAPDH, increased ROS generation, and enhanced sensitivity to apoptosis and to doxorubicin treatment (47). These findings suggest that, by maintaining proteostasis and limiting oxidative damage, CMA preserves the functional fitness of tumor cells during chronic stress, thereby facilitating the maintenance of stem-like populations.

Consistent with Hanahan’s concept of “pro-survival autophagy” as an emerging hallmark-enabling process, CMA appears to function as a lysosomal quality-control mechanism within CSCs. By selectively degrading damaged or dysfunctional proteins, CMA preserves proteostasis, metabolic homeostasis, and stress resilience, thereby enabling self-renewing tumor cells to maintain their long-term regenerative and tumor-initiating capacity under metabolic and therapeutic stress.

Together, these studies indicate that CMA contributes to replicative immortality by preserving CSC function, supporting metabolic adaptation, and enhancing resistance to therapeutic stress. Although direct evidence linking CMA to telomere maintenance remains limited, current findings support a model in which CMA enables replicative immortality indirectly by sustaining the long-term persistence of self-renewing tumor-initiating cells, thereby promoting tumor propagation, therapeutic resistance, and disease recurrence.

### 5.5 Hallmark V: inducing or accessing vasculature

Sustained tumor growth requires the formation of new blood vessels, a process primarily driven by HIF-1α and its downstream target VEGFA. Emerging evidence indicates that CMA contributes to angiogenic regulation primarily by controlling the stability of HIF-1α, the master transcriptional regulator of the hypoxic response. Under normoxic conditions, HIF-1α can be targeted for lysosomal degradation through a CMA-related mechanism involving the E3 ubiquitin ligase STUB1/CHIP, which promotes K63-linked ubiquitination and facilitates HIF-1α delivery to lysosomes (46). This CMA-dependent quality-control mechanism prevents inappropriate activation of hypoxia-responsive transcriptional programs when oxygen availability is sufficient. In contrast, during hypoxia, HIF-1α escapes lysosomal degradation, accumulates in the nucleus, and activates transcription of numerous pro-angiogenic genes, including vascular endothelial growth factor A (VEGFA), platelet-derived growth factor (PDGF), and C-X-C chemokine receptor type 4 (CXCR4), thereby promoting endothelial cell recruitment, neovascularization, and tumor vascularization (50).

### 5.6 Hallmark VI: deregulating cellular metabolism (for proliferative energetics)

Metabolic reprogramming is a hallmark of cancer that enables tumor cells to sustain rapid growth by redirecting nutrients toward biosynthesis and energy production. By selectively degrading key metabolic regulators, CMA remodels central carbon metabolism and promotes metabolic plasticity, enabling tumor cells to dynamically balance glycolysis, oxidative metabolism, and anabolic biosynthesis according to metabolic demands.

One of the earliest links between CMA and tumor metabolism was demonstrated by Kon et al. (34), who showed that CMA-mediated degradation of wild-type p53 in cancer cells relieves repression of glycolytic genes, thereby promoting glycolytic flux and metabolic adaptation. CMA also directly regulates key glycolytic enzymes (34). Lv et al. (35) reported that acetylation of pyruvate kinase M2 (PKM2) at Lys305 generates a KFERQ-like motif that targets PKM2 for CMA degradation (35). Loss of PKM2 activity slows the final step of glycolysis, leading to accumulation of upstream intermediates that are diverted into anabolic pathways, such as the pentose phosphate pathway and serine biosynthesis, thereby supporting nucleotide biosynthesis and redox balance.

CMA further modulates tumor metabolism by controlling lipid utilization. Studies from the Cuervo laboratory demonstrated that CMA selectively degrades lipid droplet coat proteins Perilipin 2 (PLIN2) and Perilipin 3 (PLIN3), enabling lipolysis and the mobilization of fatty acids for β-oxidation and energy production during metabolic stress (51, 52). Together, these findings indicate that CMA coordinates glycolysis, anabolic metabolism, and lipid utilization, thereby conferring the metabolic flexibility required for sustained tumor growth. These findings are consistent with Hanahan’s concept of metabolic plasticity and pro-survival autophagy as hallmark-enabling processes. CMA-mediated degradation of wild-type p53, PKM2, and PLIN2/3 collectively supports glycolytic, anabolic, and β-oxidation programs that fulfill the heightened biosynthetic and energetic demands of proliferating tumor cells.

Importantly, CMA-mediated metabolic regulation is highly context dependent rather than uniformly pro-tumorigenic. In cancers of mesenchymal origin, loss of CMA (via LAMP-2A knockout) paradoxically rewires the tumor metabolome toward increased anabolic and mitochondrial metabolism by enhancing TGFβR2-driven activation of glucose-6-phosphate dehydrogenase (G6PD), the rate-limiting enzyme of the pentose phosphate pathway, thereby fueling nucleotide synthesis and accelerating proliferation; pharmacological inhibition of TGFβ signaling or of mitochondrial metabolism in this setting suppresses the CMA-deficient proliferative phenotype (37). Collectively, these findings demonstrate that CMA functions as a metabolic rheostat rather than a uniformly oncogenic metabolic pathway. Depending on tumor lineage and metabolic context, CMA can either promote or restrain metabolic programs that sustain proliferation, reinforcing the broader context-dependent duality of CMA function across the hallmarks of cancer (34, 35, 37, 51, 52).

### 5.7 Emerging hallmark VII: unlocking phenotypic plasticity (and consequent functional heterogeneity)

The latest hallmarks of cancer framework includes unlocking phenotypic plasticity as an emerging hallmark, recognizing that cancer cells can dynamically alter their identity in response to intrinsic and environmental cues. This cellular flexibility enables the acquisition of stem-like and hybrid epithelial–mesenchymal states, which are increasingly associated with tumor progression, metastatic potential, and resistance to treatment (6, 7). CMA intersects with this hallmark through its regulation of EMT transcription factors, cancer stem cell maintenance programs, and TGF-β–driven cellular plasticity.

As described in earlier sections, CMA activity is enriched in GSCs relative to their differentiated counterparts, and CMA sustains stemness-associated transcriptional programs through TSC1/TSC2 degradation and constitutive mTORC1 activation (48, 49). These observations indicate that CMA supports the maintenance of stem-like cellular states, a fundamental component of phenotypic plasticity. In breast cancer, CMA regulates the EMT-promoting transcription factor Snail in a subcellular localization-dependent manner. When Snail is present in the cytoplasm, it can be recognized by HSC70 and targeted for CMA-mediated lysosomal degradation. In contrast, nuclear Snail is largely inaccessible to the CMA machinery and is therefore protected from degradation. As a result, the subcellular localization of Snail influences its stability, enabling CMA to modulate EMT and phenotypic plasticity in breast cancer cells in a context-dependent manner (53, 54). The dynamic nature of this localization gate, which is responsive to microenvironmental signals and changes in cell state, makes CMA a potentially important regulator of reversible epithelial–mesenchymal plasticity — a process now recognized as distinct from and more pervasive than classical unidirectional EMT (66).

Furthermore, the context-dependent regulation of TGFβR2 stability by CMA, as discussed in section 5.2, introduces an additional layer of control over TGF-β–driven phenotypic transitions (37). Because TGF-β signaling can promote either growth-inhibitory or pro-plasticity outcomes depending on tumor stage and microenvironmental context, CMA-mediated modulation of TGFβR2 levels places the HSC70–LAMP2A axis at a critical decision point in determining whether cells maintain epithelial identity or undergo plasticity-enabling transitions.

Emerging evidence also suggests that CMA may influence non-mutational epigenetic reprogramming, another hallmark-enabling process described by Hanahan. A seminal study by Xu et al. demonstrated that CMA is a fundamental regulator of stem cell identity by coupling metabolic remodeling to epigenetic regulation. Using embryonic stem cells, the authors showed that low CMA activity maintains pluripotency, whereas CMA activation promotes differentiation by modulating α-ketoglutarate-dependent metabolic and epigenetic pathways (26). Although these findings were obtained in normal stem cells, they establish a mechanistic framework suggesting that cancer cells may exploit similar CMA-dependent metabolic–epigenetic mechanisms to maintain stem-like phenotypes and phenotypic plasticity. Consistent with this concept, CMA has also been implicated in regulating the abundance of epigenetic modifiers such as the DNA demethylase TET3, thereby potentially influencing transcriptional programs associated with lineage plasticity and immune regulation (31). However, the contribution of this mechanism to cancer cell plasticity remains to be fully established.

Collectively, current evidence indicates that CMA contributes to phenotypic plasticity by regulating proteins that govern stemness, EMT, and TGF-β signaling. Although the mechanistic landscape is still evolving, these findings support a model in which CMA functions as a dynamic proteostatic rheostat that influences the reversibility and directionality of cancer cell state transitions, thereby contributing to intratumoral heterogeneity, therapeutic resistance, and tumor recurrence.

### 5.8 Hallmark VIII: evading immune destruction

The relationship between CMA and anti-tumor immunity is emerging as a key mechanism of immune escape, particularly through regulation of innate immune sensing, immune checkpoint stability, and the immunosuppressive tumor microenvironment. In GSCs, Li and colleagues demonstrated that MST4-driven stabilization of LAMP2A enhances CMA activity, leading to selective degradation of TET3, a DNA demethylase required for transcriptional activation of the cGAS–STING pathway. Loss of TET3 promotes epigenetic silencing of cGAS/STING, suppresses type I interferon signaling, and reduces the recruitment and activation of cytotoxic CD8^+^ T cells. Conversely, CMA inhibition restores TET3 abundance, reactivates cGAS–STING signaling, and enhances anti-tumor immunity (31).

CMA also regulates immune checkpoint abundance, although the mechanisms involved are not necessarily canonical CMA. Xu and colleagues (2024) showed that HSC70 binds PD-L1 and promotes its lysosomal degradation by disrupting the CMTM6–PD-L1 recycling axis, thereby reducing PD-L1 surface expression (55). Importantly, this process was mediated through the endosome–lysosome pathway and was shown to involve endosomal microautophagy rather than LAMP2A-dependent CMA. Thus, it should be regarded as an HSC70-associated lysosomal degradation mechanism rather than canonical CMA.

Complementing this non-canonical HSC70 pathway, recent evidence has identified STING (TMEM173) and TBK1 as direct LAMP2A-dependent CMA substrates. Their CMA-mediated lysosomal degradation dampens type I interferon signaling and anti-tumor immunity, whereas LAMP2A inhibition stabilizes STING and TBK1, enhances IFN-β production and CD8^+^ T-cell infiltration, and improves the efficacy of anti-PD-1 therapy in preclinical models. Pharmacological inhibition of LAMP2A with diclofenac further enhanced anti-PD-1 efficacy, while higher tumoral LAMP2A levels were observed in non-responders to neoadjuvant immunochemotherapy in head and neck squamous cell carcinoma, suggesting that LAMP2A may have potential as a biomarker of immunotherapy response (58).

CMA may also be therapeutically exploited to selectively redirect immune checkpoint proteins toward lysosomal degradation. In metastatic melanoma, bioinformatic analyses revealed coordinated upregulation of CMA and PD-L1 in tumors exhibiting adaptive resistance to anti-PD-1 therapy. Mechanistically, IFN-γ produced during anti-tumor immune responses induces compensatory upregulation of both PD-L1 and CMA. To exploit this adaptive state, Yan and colleagues developed a prion-like chemical inducer of proximity (SAP) that reprograms CMA by bringing PD-L1 into proximity with HSPA8/HSC70. SAP promoted CMA-dependent PD-L1 degradation and restored anti-tumor T-cell responses in both allograft and humanized patient-derived xenograft melanoma models (56). These findings establish an important distinction between inhibiting CMA globally and selectively reprogramming CMA toward the degradation of an immunosuppressive substrate such as PD-L1. The latter approach may preserve the broader proteostatic functions of CMA while selectively dismantling tumor-cell-intrinsic immune resistance (55, 57). Beyond tumor-cell-intrinsic mechanisms, CMA also regulates immune and stromal compartments within the tumor microenvironment, with effects that can either promote or restrain tumor progression. In tumor-associated macrophages (TAMs), recent studies in colorectal cancer have shown that LAMP2A is reduced in TAMs and that myeloid-specific LAMP2A deficiency increases HIF-1α abundance, enhancing secretion of VEGFA and IL-1β, thereby promoting tumor angiogenesis and tumor progression. These findings indicate that intact macrophage CMA can restrain a pro-angiogenic, pro-tumorigenic macrophage phenotype (62).

Interestingly, an earlier study in breast cancer reached a complementary but mechanistically distinct conclusion, showing that tumor-derived signals induce LAMP2A expression in TAMs and that LAMP2A-dependent degradation of PRDX1 and CRTC1 promotes pro-tumorigenic macrophage activation (67). This apparent context dependence suggests that the consequences of macrophage CMA may vary according to tumor type, inflammatory environment, and the specific CMA substrates engaged.

CMA also governs the suppressive function of regulatory T cells (Tregs). Recent work demonstrated that CMA activity increases upon Treg activation and is required for optimal Treg suppressive function. Treg-specific LAMP2A deletion impaired Treg-mediated immune suppression and, importantly, enhanced anti-tumor immune responses in a syngeneic tumor model, indicating that CMA supports the immunosuppressive activity of Tregs within the tumor microenvironment (59, 60).

A further stromal contribution to CMA-mediated immune regulation arises from tumor-associated pericytes. In glioblastoma, tumor-cell contact induces aberrant CMA upregulation in peritumoral pericytes, converting them into a pro-tumorigenic and immunomodulatory mesenchymal stromal-like phenotype that facilitates tumor progression. The CMA-modulating phosphopeptide P140 reverses this aberrant CMA activation, disrupts pericyte–tumor cell interactions, and induces a tumor-toxic secretome. MAPT/tau, a CMA substrate, was identified as a major component of this secretome, and perivascular MAPT/tau accumulation was proposed as a biomarker of P140 response (68). Evidence directly establishing a CMA-dependent mechanism in cancer-associated fibroblasts remains limited. Therefore, CAFs should currently be considered a potential rather than established compartment of CMA-mediated immune regulation.

Collectively, these mechanisms position CMA and CMA-associated chaperone/lysosomal systems within Hanahan’s immune-evasion circuitry, where they regulate both tumor-cell-intrinsic immune sensing and the functional state of immune and stromal cells in the tumor microenvironment. CMA can suppress anti-tumor immunity by promoting degradation of TET3, STING, and TBK1, while HSC70-associated lysosomal pathways can regulate PD-L1 turnover. At the same time, CMA in macrophages, Tregs, and pericytes can reshape the tumor microenvironment in a context-dependent manner.

Therapeutically, these findings suggest two potential strategies for targeting CMA: inhibiting CMA to preserve innate immune sensors such as STING and TBK1, and selectively redirecting CMA to degrade immunosuppressive proteins such as PD-L1. By enhancing innate immune signaling and restoring anti-tumor T-cell activity, these approaches suggest that CMA-dependent lysosomal degradation could be a promising therapeutic target for overcoming both innate and adaptive immune resistance (56, 58).

Overall, these findings indicate that CMA can promote tumor immune escape through several context-dependent mechanisms, including suppression of innate immune sensing, regulation of immune checkpoint proteins, and modulation of immune and stromal cells in the tumor microenvironment. Importantly, not all HSC70-dependent lysosomal degradation pathways are canonical CMA. Therefore, distinguishing LAMP2A-dependent CMA from other HSC70-mediated lysosomal pathways is essential for accurately understanding the role of CMA in tumor immunity.

### 5.9 Hallmark IX: activating invasion and metastasis

Metastasis is a multistep process involving local invasion, intravasation, survival in circulation, and colonization of distant organs. CMA contributes to the regulation of metastasis by selectively degrading proteins involved in epithelial–mesenchymal transition (EMT), cell migration, and signaling pathways that govern metastatic dissemination. Importantly, current evidence indicates that CMA can exert both pro-metastatic and anti-metastatic effects, depending on tumor lineage, cellular context, and the specific substrates targeted for degradation.

At the primary tumor site, CMA can promote invasion by degrading negative regulators of migratory signaling. In papillary thyroid carcinoma, CMA-mediated degradation of PPARγ activates the SDF1/CXCR4 axis, thereby enhancing directional migration and lymph node metastasis (61). This finding illustrates how CMA-mediated removal of a metastasis-suppressive regulator can promote tumor cell motility and dissemination.

Conversely, CMA can also restrain tumor invasion and metastasis in a context-dependent manner. Using isoform-specific LAMP-2A knockout models, Zhou and colleagues demonstrated that CMA deficiency promotes widespread tumor cell dissemination, vascular invasion, and metastasis, and that LAMP-2A expression is consistently reduced in metastatic lesions compared with matched primary tumors from the same patients (37). Mechanistically, CMA suppresses EMT and metastatic dissemination by selectively degrading key pro-metastatic signaling proteins, including TGFβR2, thereby limiting TGF-β signaling. Loss of the CMA receptor LAMP-2A stabilizes these proteins, resulting in sustained TGF-β signaling, enhanced EMT, increased cellular plasticity, and metastatic spread. Conversely, TGF-β signaling suppresses LAMP-2A expression, establishing a reciprocal feedback loop between CMA and TGF-β signaling that influences epithelial–mesenchymal state transitions and metastatic potential (37). This anti-metastatic, tumor-suppressive function of CMA in mesenchymal tumors is consistent with observations in other tumor types, including hepatocellular carcinoma (39) and IDH1-wild-type glioblastoma (38), where reduced CMA activity is associated with more aggressive tumor behavior. Together, these findings support the concept that CMA can function either as a metastasis-promoting or metastasis-suppressive pathway, depending on tumor lineage and the substrates under its control.

Emerging data further suggest that CMA may interact with other protein-degradation systems, including the ubiquitin–proteasome pathway, in regulating EMT-associated transcription factors such as Snail. The subcellular localization of Snail appears to be particularly important: cytoplasmic Snail can be targeted for HSC70-dependent lysosomal degradation, whereas nuclear Snail is relatively protected from CMA-mediated turnover. This provides a potential mechanism through which CMA may influence the reversibility of EMT and the plasticity of metastatic tumor cells. However, the extent to which this mechanism contributes directly to metastatic dissemination remains to be fully established (53, 54).

Collectively, these findings indicate that CMA regulates invasion and metastasis through a context-dependent network of substrates and signaling pathways. Rather than functioning as a uniformly pro-metastatic pathway, CMA can either promote tumor cell migration and dissemination or restrain EMT and metastatic progression, depending on tumor lineage, substrate availability, and the tumor microenvironment. This bidirectional behavior further emphasizes the broader context-dependent role of CMA across the hallmarks of cancer.

## 6 An overall view: CMA as a multi-hallmark regulator and translational implications

The evidence reviewed in the preceding sections collectively establishes CMA as more than a selective degradative pathway, but an important proteostatic regulator. Its context-dependent dysregulation in cancer cells enables the coordinated engagement of multiple hallmark capabilities (15, 34, 37–39). This is a conceptually important distinction because most oncogenic driver mutations, such as activating mutations in KRAS or PIK3CA, loss-of-function alterations in TP53 or RB1, and epigenetic silencing of tumor suppressor genes, typically exert their primary influence over one or a limited number of downstream signaling pathways. CMA, by contrast, acts at the post-translational level, enabling cancer cells to dynamically rewire a remarkably broad spectrum of multiple functional programs through selective substrate degradation, without requiring genetic alterations in the CMA pathway itself.

It is important to stress, that the strength of evidence supporting this model is not uniform across different tumor types. The pro-tumorigenic role of CMA in gastric, colorectal, and glioblastoma stem cell models is supported by cell-line studies, patient-derived xenograft models, and TCGA-based correlative analyses (31, 47–49). In contrast, the tumor-suppressive role of CMA in hepatocellular carcinoma and mesenchymal tumors is currently supported primarily by LAMP2A knockout mouse models and matched patient tissue validation rather than large clinical cohorts (37, 39). This difference in evidentiary maturity should be considered when interpreting the therapeutic implications of CMA across different cancer settings.

A further layer of context dependence relates to tumor stage. Several lines of evidence suggest that CMA may exhibit a biphasic role during tumorigenesis. During early oncogenesis, CMA functions as a tumor-suppressive quality-control mechanism by clearing oxidatively damaged proteins and limiting chronic inflammation-driven mutagenesis. However, once malignant transformation is established, tumor cells appear to co-opt CMA to maintain stress tolerance, proliferative signaling, and survival. This temporal switch resembles the well-established dual role of macroautophagy in tumor suppression and tumor promotion (8, 9, 34).

The diverse substrate repertoire identified across multiple studies highlights the broad regulatory role of CMA in cancer (summarized in **Table 1**). The HSC70–LAMP2A translocation complex mediates the selective degradation of a diverse set of proteins with distinct biological functions, including wild-type p53 (thereby abrogating tumor suppression and enabling metabolic reprogramming), TSC1/TSC2 (thereby constitutively activating mTORC1 to sustain proliferation and stemness), PUMA/BBC3 (suppressing intrinsic apoptosis), CHK1 (modulating the DNA damage checkpoint), TET3 (epigenetically silencing the cGAS–STING pathway and enabling immune evasion), PPARγ (activating the SDF1/CXCR4 metastatic axis), and TTC28 (promoting chromosomal instability).

What is striking is not only the number of CMA substrates but also their functional non-redundancy and, in several cases, opposing directionality opposing functional consequences. Each substrate maps to a distinct hallmark of cancer and, depending on the tumor lineage, its degradation either dismantles tumor-suppressive barriers or removes constraints on malignancy. This is, in effect, a form of oncogenic convergence, where diverse hallmark-enabling or hallmark-restraining outcomes arise from the same core degradation machinery.

These findings suggest that CMA upregulation functions as a proteostatic amplifier rather than an initiating oncogenic event. By selectively degrading multiple regulatory proteins, CMA accelerates the acquisition of hallmark capabilities in cells that have already undergone initiating genetic alterations. In tumor-suppressive contexts, the same mechanism may instead restrain hallmark acquisition. In pro-tumorigenic settings, initiating mutations, such as KRAS activation or TP53 loss, create selective pressure that favors cells with elevated CMA activity because these cells can more rapidly consolidate a fully malignant phenotype by degrading residual tumor-suppressive proteins that would otherwise restrain further progression.

Conversely, CMA upregulation may itself promote evolutionary selection by enhancing chromosomal instability through TTC28 degradation. This increases tumor cell mutational diversity, accelerates Darwinian selection, and facilitates the emergence of therapy-resistant clones. Consequently, CMA may establish a CMA–genomic instability feed-forward loop, in which CMA hyperactivation both drives and reinforces the evolving mutational landscape of the tumor (40, 44).

Furthermore, CMA engages not only the six original hallmarks of the original Hanahan framework, but also the more recently defined enabling characteristics and emerging hallmarks introduced in the 2022 and 2026 Hallmarks of Cancer updates. The demonstration that CMA targets CHK1 and TTC28 links it to genome instability; its regulation of HMGB1 clearance and NF-κB stability connects it to tumor-promoting inflammation; and its modulation of Snail stability, TGFβR2 turnover, and GSC identity programs links CMA to phenotypic plasticity (26–28, 40, 44, 53, 54). The 2026 update further emphasizes systemic dimensions of tumor evolution, particularly aging and obesity, to which CMA is closely linked. CMA activity declines progressively with age due to destabilization of LAMP2A within lysosomal lipid microdomains (29, 69). This age-related decline may diminish either the pro-tumorigenic CMA flux that supports established tumors or the tumor-suppressive quality-control function of CMA during early oncogenesis, depending on tissue context. Together, these observations provide a mechanistic link between aging and the increasing incidence of cancer in older populations.

Similarly, obesity-associated metabolic stress and chronic low-grade inflammation are known triggers of both macroautophagy and CMA. Because CMA governs lipid droplet turnover through PLIN2/PLIN3 degradation (51, 52), obesity-driven lipid overload may directly alter CMA substrate flux at the adipose–tumor interface. Whether this influences tumor evolution remains largely unexplored and represents an important knowledge gap within the updated Hallmarks of Cancer framework (7).

These observations also have important translational implications. As a context-dependent proteostatic regulator, CMA may drive tumor evolution, therapeutic resistance, and metastatic progression by promoting cellular adaptability and genomic instability in some settings, while restraining tumorigenesis in others. Consequently, targeting CMA offers a unique opportunity to simultaneously disrupt multiple hallmark capabilities. However, its clinical translation is constrained by substantial druggability challenges. LAMP2A performs essential homeostatic functions in normal tissues, including hepatocytes, T cells, and aged neurons. Therefore, systemic pharmacological inhibition may result in off-target toxicity, including impaired T-cell function and accelerated proteotoxic stress in non-malignant cells (20, 29). Tumor-selective strategies are therefore a priority, and the CMA-rewiring chemical-inducer-of-proximity approach that redirects PD-L1 to CMA-mediated degradation illustrates one route to achieving substrate selectivity without global CMA inhibition (55). However, validated predictive biomarkers capable of prospectively identifying tumors that depend on CMA gain or CMA loss are still lacking. This remains a major barrier to the rational clinical deployment of CMA-targeted therapies. Nevertheless, CMA activity may serve as a biomarker of tumor aggressiveness and treatment response, supporting patient stratification and precision oncology while strengthening the rationale for CMA-targeted therapeutic interventions once predictive biomarkers become available.

## 7 Conclusion and future directions

CMA functions as a multistage cancer-hallmark regulator that coordinates tumor cell adaptation through selective protein degradation. Unlike pathways that predominantly affect a single signaling axis, CMA simultaneously influences proliferation, survival, metabolism, immune evasion, phenotypic plasticity, and metastatic behavior by remodeling the cancer proteome at the post-translational level. This broad functional reach positions CMA as a central integrator of malignant fitness and helps explain why its activity is frequently elevated across diverse tumor types. Critically, this multi-hallmark regulatory reach is bidirectional rather than uniformly oncogenic: emerging genetic evidence from hepatocellular carcinoma, mesenchymal tumor metastasis, and IDH1-wild-type glioblastoma demonstrates that CMA loss, not CMA gain, can drive tumorigenesis in specific tissue contexts, positioning CMA as a dual-function proteostatic node whose net effect on malignancy is dictated by tumor lineage, driver mutation background, and substrate availability rather than by a single universal mechanism (37–40).

These insights establish CMA as a promising target for cancer therapy while also revealing important knowledge gaps. Future studies should define the molecular determinants of substrate selectivity, identify tumor contexts that are most dependent on CMA, and elucidate its interplay with other proteostasis and stress-response pathways. The development of reliable biomarkers of CMA activity and tumor-selective pharmacological modulators, together with validation in clinically relevant models, will be critical for translating these discoveries into precision oncology. A deeper understanding of the CMA regulatory network will ultimately determine whether targeting this pathway can provide durable therapeutic benefit across multiple cancer types. A central priority for future work is to resolve the molecular basis of CMA’s tissue- and lineage-specific switch between tumor-promoting and tumor-suppressive function — for instance, why LAMP-2A loss is oncogenic in hepatocytes and mesenchymal tumors but CMA activation is oncogenic in GSCs—since this determinant will dictate whether CMA-directed therapy should employ activators or inhibitors in a given tumor. Concrete, testable avenues include: (i) developing small-molecule or peptide inhibitors of LAMP-2A homo-oligomerization (which is required for substrate translocation) and evaluating their synthetic-lethal effects specifically in STAG2-wild-type versus STAG2-mutant, or KRAS-mutant versus KRAS-wild-type, tumor backgrounds; (ii) using KFERQ-motif proteomic screens to define tumor-type-specific CMA substrate hierarchies that could explain why substrate competition favors degradation of tumor suppressors in some cancers and oncoproteins in others; (iii) testing scheduled, treatment-window-restricted CMA inhibition (rather than continuous inhibition) in combination with chemotherapy to counteract drug-induced upregulation of CMA-mediated PUMA/CHK1/p53 clearance; and (iv) validating LAMP2A tumor expression as a predictive biomarker for immune checkpoint inhibitor response in prospective cohorts, building on the retrospective head-and-neck squamous cell carcinoma association already reported.
